# Magnetic resonance imaging tracing of superparamagnetic iron oxide nanoparticle–labeled mesenchymal stromal cells for repairing spinal cord injury

**DOI:** 10.4103/NRR.NRR-D-24-00431

**Published:** 2024-10-22

**Authors:** Xiaoli Mai, Yuanyuan Xie, Zhichong Wu, Junting Zou, Jiacheng Du, Yunpeng Shen, Hao Liu, Bo Chen, Mengxia Zhu, Jiong Shi, Yang Chen, Bing Zhang, Zezhang Zhu, Bin Wang, Ning Gu

**Affiliations:** 1 Department of Radiology, Nanjing Drum Tower Hospital, Affiliated Hospital of Medical School, Nanjing University, Nanjing, Jiangsu Province, China; 2 Clinical Stem Cell Center, Nanjing Drum Tower Hospital, Affiliated Hospital of Medical School, Nanjing University, Nanjing, Jiangsu Province, China; 3 Department of Spinal Surgery, Nanjing Drum Tower Hospital, Affiliated Hospital of Medical School, Nanjing University, Nanjing, Jiangsu Province, China; 4 State Key Laboratory of Bioelectronics and Jiangsu Key Laboratory of Biomaterials and Devices, School of Biological Sciences and Medical Engineering, Southeast University, Nanjing, Jiangsu Province, China; 5 Laboratory of Image Science and Technology, Southeast University, Nanjing, Jiangsu Province, China; 6 Department of Neurosurgery, Nanjing Drum Tower Hospital, Affiliated Hospital of Medical School, Nanjing University, Nanjing, Jiangsu Province, China; 7 Institute of Materials Science and Devices, School of Materials Science and Engineering, Suzhou University of Science and Technology, Suzhou, Jiangsu Province, China; 8 Department of Pathology, Nanjing Drum Tower Hospital, Affiliated Hospital of Medical School, Nanjing University, Nanjing, Jiangsu Province, China; 9 Nanjing Key Laboratory for Cardiovascular Information and Health Engineering Medicine, Institute of Clinical Medicine, Nanjing Drum Tower Hospital, Affiliated Hospital of Medical School, Nanjing University, Nanjing, Jiangsu Province, China

**Keywords:** acute spinal cord injury, diffusion tensor imaging, dynamic migration, mesenchymal stromal cells, neural function, neuronal regeneration, quantitative susceptibility mapping, repairability, ruicun, superparamagnetic iron oxide nanoparticle

## Abstract

Mesenchymal stromal cell transplantation is an effective and promising approach for treating various systemic and diffuse diseases. However, the biological characteristics of transplanted mesenchymal stromal cells in humans remain unclear, including cell viability, distribution, migration, and fate. Conventional cell tracing methods cannot be used in the clinic. The use of superparamagnetic iron oxide nanoparticles as contrast agents allows for the observation of transplanted cells using magnetic resonance imaging. In 2016, the National Medical Products Administration of China approved a new superparamagnetic iron oxide nanoparticle, Ruicun, for use as a contrast agent in clinical trials. In the present study, an acute hemi-transection spinal cord injury model was established in beagle dogs. The injury was then treated by transplantation of Ruicun-labeled mesenchymal stromal cells. The results indicated that Ruicun-labeled mesenchymal stromal cells repaired damaged spinal cord fibers and partially restored neurological function in animals with acute spinal cord injury. T2*-weighted imaging revealed low signal areas on both sides of the injured spinal cord. The results of quantitative susceptibility mapping with ultrashort echo time sequences indicated that Ruicun-labeled mesenchymal stromal cells persisted stably within the injured spinal cord for over 4 weeks. These findings suggest that magnetic resonance imaging has the potential to effectively track the migration of Ruicun-labeled mesenchymal stromal cells and assess their ability to repair spinal cord injury.

## Graphical abstract

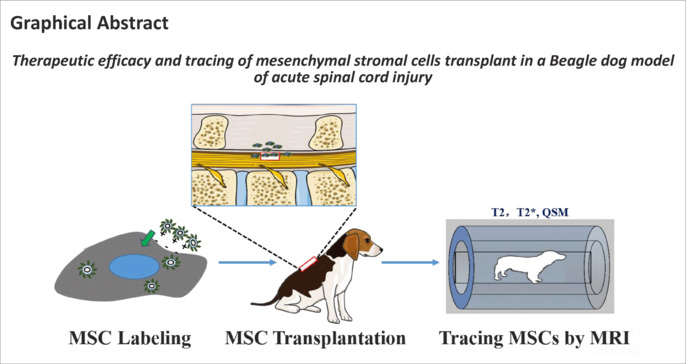

## Introduction

Spinal cord injury (SCI) results in complete or incomplete motor, sensory, and sphincter dysfunction of the area below the injured segment (Talbott et al., 2019), and accounts for tens of billions of dollars in medical expenses (Shao et al., 2019; Guan et al., 2023). Protecting residual nerve function and regenerating neural circuits are new treatment strategies in SCI (Shao et al., 2019; Montoto-Meijide et al., 2023). Mesenchymal stromal cell (MSC) transplantation is an efficient and promising therapeutic approach in the treatment of many systemic and diffuse diseases (Assinck et al., 2017; Sun et al., 2023; Norte-Muñoz et al., 2024). Currently, a search on clinicaltrials.gov indicates that more than 650 clinical trials of MSC therapies have been registered worldwide, including 60 studies on the use of MSCs in SCI. However, the metabolic process of transplanted MSCs cannot be studied in the human body as is done for drug therapies, which poses a significant challenge to these studies. This has led to an incomplete understanding of the biology of transplanted MSCs *in vivo*, including cell viability, distribution, migration, and fate in humans. Therefore, it is necessary and urgent to develop noninvasive methods to dynamically monitor the survival, distribution, and migration of transplanted MSCs in recipient patients.

Traditional cell tracking methods require that experimental animals be killed to trace the transplanted cells, so these methods cannot be used in recipient patients. Magnetic resonance imaging (MRI) is an ideal tracking technique for labeled cells using superparamagnetic iron oxide nanoparticles (SPIONs) (Bulte et al., 2002; Unger, 2003; Chen et al., 2024) due to its advantageous characteristics such as noninvasiveness, high resolution, repetitive operations, and good tissue contrast (Thorek et al., 2006; Wang et al., 2020; Li et al., 2023). SPIONs are paramagnetic transition metals that produce low signals in T1-, T2-, and T2*-weighted images and short T1, T2, and T2* relaxation times (Kuhlpeter et al., 2007; Girard et al., 2012; Huang et al., 2024; Li et al., 2023). Ferumoxytol is a U.S. Food and Drug Administration-approved injectable form of iron for the treatment of iron deficiency anemia in patients with renal failure. Ferumoxytol has recently been used as a molecular probe to investigate cellular dynamics using MRI by several research groups (Daldrup-Link et al., 2017; Nejadnik et al., 2018; Theruvath et al., 2019). Ruicun (RC) is a generic form of Ferumoxytol that was approved by the National Medical Products Administration of China in 2016 for its use as a clinical trial drug. Using safe contrast agents, MRI can noninvasively capture cell implanting location, migration, and distribution in real time to track the outcomes of stem cell transplantation.

Quantitative susceptibility mapping (QSM) is an advanced technique in MRI to quantify the magnetic susceptibility of substances (Barbosa et al., 2015; Acosta-Cabronero et al., 2016). It has been used to estimate iron accumulation *in vivo*, and a positive correlation between magnetic susceptibility and iron concentration has been reported (Wang and Liu, 2015; Sharma et al., 2017). Ultrashort echo time (UTE) sequences measured R2*(1/T2*) values of paramagnetic signal sources with high iron concentration. Improvement in signal detection and phase measurement using UTE in the QSM (UTE-QSM) may allow for more accurate estimates of magnetic susceptibility when T2* is greatly reduced by high iron nanoparticle concentration (Lu et al., 2018). Therefore, UTE-QSM might be a promising technique to detect RC-labeled MSCs (RC-MSCs) distribution *in vivo*. In acute SCI (ASCI), the most critical pathophysiological changes are axonal damage and demyelination of white matter fiber bundles (Brennan et al., 2013). Diffusion tensor imaging (DTI) is useful to determine the dispersion of water molecules after ASCI by quantitative measurements and fiber bundle tracer imaging (diffusion tensor tractography, DTT), which may help evaluate the integrity of spinal fibers after MSC therapy (Wang-Leandro et al., 2018; Zhu et al., 2021).

This study used a semi-transverse ASCI model in beagle dogs and RC labeling of MSCs *in vitro*. We hypothesized that labeled and unlabeled MSCs would have a similar degree of spinal nerve functional recovery. Additionally, we hypothesized that multiparameter MRI, combining postprocessing technology, histopathology, and immunohistochemistry, would be useful for evaluating the therapeutic effect of MSCs in ASCI and quantitatively analyzing the dynamic behaviors of transplanted MSCs, such as distribution, migration, and differentiation in the host within 28 days after transplantation.

## Methods

### Culture, labeling, and identification of mesenchymal stromal cells

MSCs were derived from neonatal umbilical cord tissue and isolated using the tissue explant culture method (Nguyen et al., 2022). All samples used for MSC isolation in this study were obtained from donors at Nanjing Drum Tower Hospital (Nanjing, Jiangsu Province, China). Informed consent was obtained from each subject prior to participation with the approval of the Institutional Review Board (No. 2017-161-08; November 29, 2023). This study followed the principles laid out in the 2021 ISSCR Guidelines for Stem Cell Research and Clinical Translation (Lovell-Badge et al., 2021). Culture and identification of MSCs were performed according to the procedure in our previous studies (Xie et al., 2020, 2021). MSC labeling with RC was performed as described in our previous study (Xie et al., 2019). Prussian blue (PB; G1426; Solarbio, Beijing, China) staining was used to detect iron oxide in MSCs after RC labeling, following the procedure in our previous studies (Mai et al., 2009; Xie et al., 2019). The RC-MSCs were digested into single cells and seeded on a cell slide. The slides were incubated with anti-human CD90 (1:50, Abcam, Cambridge, MA, USA, Cat# ab133350, RRID: AB_11155503).

### EdU cell proliferation assay

The proliferation level of MSCs was detected using the EdU assay kit (C00052, Ribobio, Guangzhou, Guangdong, China) following the manufacturer’s instructions. Briefly, MSCs from different treatment groups were seeded onto culture plates and incubated until they reached 60%–70% confluence. EdU reagent (50 μM) was then added, and the cells were incubated for 2 hours. After incubation, 4ʹ,6-diamidino-2-phenylindole (DAPI, Abcam, Cat# ab104139) was added for nuclear staining. Fluorescent microscopy was used to observe and capture images of the cells. The proportion of EdU-positive cells was then calculated (Zhou et al., 2023).

### Animal surgery and cell implantation

Adult female beagle dogs (18 months old, weighing 12–14 kg) provided by Yizheng Anlimu Biotechnology Co., Ltd. (Yangzhou, Jiangsu Province, China; animal license No. SCXK (Su) 2016-0005) were housed in a temperature- and humidity-controlled environment for at least 7 days before surgery. All experiments were performed in accordance with the Guide for the Care and Use of Laboratory Animals (National Research Council, 2011) and were approved by the Animal Care and Use Committee of Nanjing Drum Tower Hospital, Affiliated Hospital of Medical School, Nanjing University on June 8, 2018 (approval No. 2018060002).

**Figure 1:**
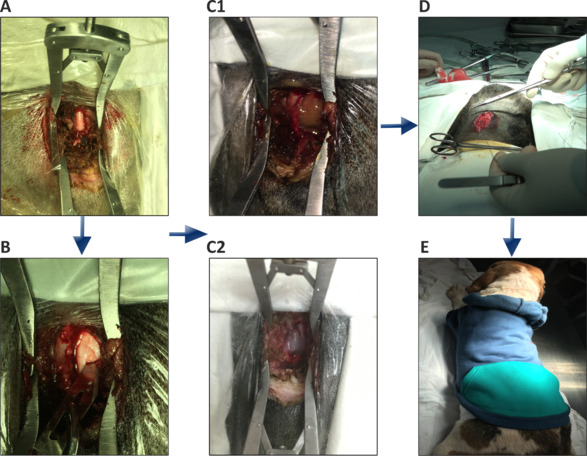
ASCI surgery at T8 spinal cord segment and treatment. (A) A midline incision approximately 5 cm long was made at the T8 level in beagle dogs to expose the spinal cord tissue. (B) The right side of the spinal cord was partially transected by an approximately 0.5-cm cut with a scalpel to establish the ASCI beagle dog model. (C) The cell therapy groups received transplants of RC-MSCs-CMDiI (C1, *n* = 15) or MSCs-CMDiI (C2, *n* = 15). (D) Absorbable sutures were used to close the wound at the site of the spinal cord injury in the dogs. (E) Postoperatively, the wounds were dressed to prevent infection. ASCI: Acute spinal cord injury; MSCs-CMDiI: mesenchymal stromal cells labeled by CMDiI; RC-MSCs-CMDiI: mesenchymal stromal cells labeled by Ruicun and CMDiI.

With 12-hour fasting before surgery, the animals were administered atropine sulfate (0.1 mg/kg; Harbin Pharmaceutical Group Sanjing Pharmaceutical Co., Ltd., Harbin, Heilongjiang Province, China) to decrease bronchial and salivary secretions and were anesthetized with tiletamine zolazepam (Zoletil; Virbac S.A., Carros, France) at a dosage of 10 mg/kg. Anesthesia was maintained by constant intravenous administration of propofol (0.20 mg/kg/min; Guangdong Jiabo Pharmaceutical Co., Ltd., Qingyuan, Guangdong Province, China), and discontinuous intravenous fentanyl (0.002 mg/kg/30 min; Jiangsu Nhwa Pharmaceutical Co., Ltd., Xuzhou, Jiangsu Province, China) was used during surgery. A continuous supply of oxygen was provided through an endotracheal tube, and the ventilator was set at approximately 15 respirations per minute during the surgery. Under aseptic conditions, a midline incision of approximately 5 cm was made at the level of T8 (**Figure 1A**). A 0.5-cm cut was made in the right side of the spinal cord to simulate the injury (**Figure 1B**).

All animals were randomly allocated into three groups to receive different transplantation treatments 4 hours after the SCI. During those 4 hours, we performed monitoring, analgesia, administration of the immunosuppressant dexamethasone (0.08 mg/kg; Shanghai Modern Hasen Pharmaceutical Co., Ltd., Shanghai, China) and fluid infusion on the dogs undergoing surgery. The control group received normal saline after injury (*n* = 15), the MSCs group received 1 × 10^7^ MSCs-CMDiI (*n* = 15), and the RC-MSCs group received 1 × 10^7^ RC-MSCs-CMDiI (*n* = 15) (**Figure 1C1** and **C2**). CMDiI is one of the most commonly used cell membrane fluorescent probes and emits orange-red fluorescence. CMDiI is a lipophilic membrane dye, and it can diffuse laterally to gradually stain the whole cell membrane after entering the cell (Yang et al., 2021). The RC-MSCs labeled with iron or unlabeled MSCs were separately digested into single cells, resuspended in 20 μg/mL CMDiI dye, and incubated at 37°C for 15 minutes and then at 4°C for 15 minutes. The resulting cells were CMDiI-labeled cells (RC-MSC-CMDiI cells or MSCs-CMDiI). Finally, the dura was sutured with nonabsorbable 4/0 silk sutures, paraspinal muscles were sutured separately with 0 silk sutures, and skin with 2-0 silk sutures (**Figure 1D**). After transplantation surgery, the wounds were dressed to protect from infection (**Figure 1E**).

### Behavioral assessment

The Texas Spinal Cord Injury Score (TSCIS; a 10-point scoring system) was used to evaluate functional motor recovery in the dogs with ASCI (Levine et al., 2009). TSCIS is a comprehensive grading system designed to assess the severity of SCI and its impact on functional abilities. This scoring system evaluates motor and sensory functions, reflexes, and autonomic functions, providing a standardized method to quantify the extent of the injury and predict potential recovery outcomes. Two individuals who were blinded to the experimental conditions recorded a TSCIS for each beagle at weeks 1, 2, 4, 6, 8, and 10 after the surgical transplantation of MSCs. During the assessment, each beagle was evaluated for automatic or nonautomatic movements of the hindlimbs after moving them to an open area.

### Magnetic resonance imaging

Each dog underwent MRI of the spinal cord at 3, 7, 14, 21, 28, 35, 42, 56, 70, 91, and 105 days after the surgical implantation procedure. Multiparameter MRI was performed using an Ingenia 3.0 T CX MRI system (Philips Healthcare, Amsterdam, the Netherlands) and a 64-channel abdominal coil. MRI included T1WI with turbo spin echo sequence (repetition time [TR]/echo time [TE]: 590 ms/8 ms, flip angle: 90°, field of view: 150 mm × 300 mm, matrix size: 300 × 150, section thickness: 2 mm), T2WI with mDixon turbo spin echo sequence (TR/TE: 2500 ms/100 ms, flip angle: 90°, field of view: 150 mm × 300 mm, matrix size: 160 × 350, section thickness: 2 mm), DTI with single shot spin echo-planar imaging (TR/TE: 5549 ms/71 ms, flip angle: 90°, field of view: 150 mm × 200 mm, matrix size: 100 × 98, diffusion sensitive gradient directions: 15, B-value: 0, 800 mm^2^/s, section thickness: 2 mm), T2 mapping with multi-echo turbo spin-echo sequence (TR/TE: 700 ms/10 ms, 16 ms, 22 ms, 28 ms, and 34 ms; flip angle: 90°; field of view: 130 mm × 130 mm; matrix size: 132 × 129; section thickness: 2 mm), and QSM based on four-echo fast field echo sequence with an ultrashort echo (TR/TE: 10 ms/0.19 ms, 2.5 ms, 4.8 ms, and 7.1 ms; flip angle: 10°; field of view: 130 mm × 130 mm; matrix size: 132 × 132; section thickness: 4 mm). The ∆TE on T2 mapping and UTE-QSM analysis was 6 ms and 2.3 ms, respectively.

### Magnetic resonance imaging data analysis

#### Diffusion tensor imaging data processing and diffusion tensor tractography image reconstruction

Using the Philips postprocessing workstation (IntelliSpace Portal, ISP, Philips Healthcare), the region of interest (ROI) was sketched on the DTI images with the axial T2WI as the reference. The ROI included all of the spinal cord tissue at the level of the injury while avoiding hemorrhagic and edematic areas. The quantitative parameter, fractional anisotropy (FA), was calculated from DTI data, and the fiber tracts were visually traced using DTT. Each ROI was measured three times and averaged.

#### T2 mapping data processing

T2 mapping was generated automatically with the host scanner (Ingenia 3.0T CX, Philips Healthcare), and T2 relaxation times were measured in each animal in operator-defined ROIs with hypo-signal by a radiologist with 6 years of experience.

#### Quantitative susceptibility mapping reconstruction

The multi-echo fast field echo images from the UTE sequence included complex MRI data of each echo time and T2* mapping. The in-phase echoes were acquired for QSM analysis to remove the effects of chemical shifts and to improve the signal-to-noise ratio. QSM data processing solved the magnetic susceptibility distribution in reverse from the phase change, mainly based on complex MRI data of different echoes (Acosta-Cabronero et al., 2017). The reconstruction consisted of the following four steps. (1) Field map fitting: the field map fitting was calculated with the complex MRI data collected from a multi-echo fast field echo sequence using a nonlinear least square fitting algorithm. (2) Phase unwrapping: the continuous Laplacian-based unwrapping algorithm (Acosta-Cabronero et al., 2017) was used for phase unwrapping to generate a smooth phase map. (3) Background field removal: the projection onto the dipole field algorithm was used to acquire a local field map (Jang et al., 2020). (4) Magnetic susceptibility inversion: the local field map was input to the morphology-enabled dipole inversion QSM algorithm to estimate the final susceptibility map (Jang et al., 2020). The T2* relaxation time was acquired from T2* mapping, which was generated automatically. The T2* relaxation time of the same layer of muscle tissue was measured, and rT2* = T2* relaxation time of ROIs/T2* relaxation time in the muscle tissue at the same level.

### Histological and immunofluorescence analysis

For immunocytochemistry, anesthesia was induced on the beagles with tiletamine zolazepam (Zoletil; Virbac) at a dosage of 10 mg/kg. After anesthesia induction, euthanasia was carried out by intravenous injection of propofol (2 mg/kg) to induce overdose. Beagles were sacrificed after MR scanning at day 3, week 1, and weeks 2, 3, 4, 5, 7, 9, 11, 13, and 15 of surgical transplantation. Spinal cord segments from the T7–T9 region were collected. Adjacent tissue sections were double stained with PB and eosin (C0109; Beyotime, Shanghai, China) for general observation of intracellular and extracellular matrix iron deposition characteristics. For immunocytochemistry, the slides were incubated with the following primary antibodies: anti-human-CD90 (1:50; Abcam, Cat# ab133350) and anti-dog-Tuj-1 (1:500; Abcam, Cat# ab7751, RRID: AB_306045), both at 4°C overnight. CD90 is an MSC membrane surface protein, which can be tracked to assess cell viability (Saalbach and Anderegg, 2019). Tuj-1 is a neuronal marker, which can be used to assess the ability of the spinal cord to regenerate after the transplantation of MSCs (or RC-MSCs) following injury (Liu et al., 2022; Wang et al., 2024). Subsequently, the samples were incubated with secondary antibodies (Alexa Fluor 488 donkey anti-mouse, 1:500, Thermo Fisher Scientific, Carlsbad, CA, USA, Cat# A-11011, RRID: AB_143157; Alexa Fluor 488 goat anti-rabbit, 1:500, Thermo Fisher Scientific, Cat# A-11034, RRID: AB_2576217). Cell nuclei were stained with DAPI (Abcam, ab104139), and images were captured under a Leica DMi8 confocal microscope (Leica Microsystems, Wetzlar, Hessen, Germany).

### Statistical analysis

GraphPad Prism version 10.0.0 for Windows (GraphPad Software Inc., Boston, MA, USA, www.graphpad.com) was used for statistical analysis. All data are presented as mean ± standard deviation. Levene’s test for homogeneity was used to determine the normal distribution and variance of TSCIS, FA, T2, T2*, rT2*, and QSM. Parametric methods were used for normally distributed data. Nonparametric methods, such as the Mann–Whitney *U* test with Dunn’s *post hoc* analysis, were used for data that were not normally distributed. For data satisfying the homogeneity of variance criteria, independent samples *t*-tests or two-way analysis of variance with Tukey’s *post hoc* test or least significant difference test were used. Pearson correlation analysis was conducted to evaluate the relationships between FA and TSCIS and between T2*, rT2*, and QSM. An *R* > 0 indicates a positive correlation, whereas an *R* < 0 indicates a negative correlation. A *P*-value < 0.05 was considered statistically significant.

## Results

### Ruicun and CMDiI labeling efficiency mesenchymal stromal cell

**Figure 2:**
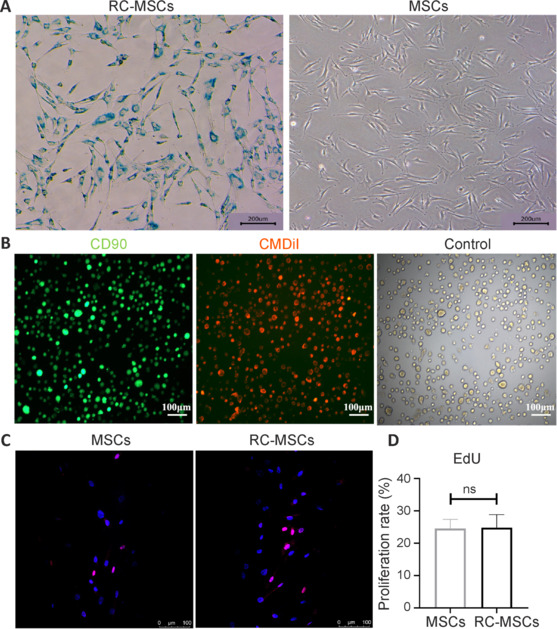
The identification of RC-MSCs-CMDiI. (A) In labeled cells, PB staining revealed the intracellular accumulation of iron nanoparticles, indicating that nearly all of the cells were labeled with RC. The left panel shows the PB-stained image and the right panel shows the unstained control image. Scale bars: 200 μm. (B) Representative images of immunostaining for CD90 (MSCs: Alexa Fluor 488, green) and CMDiI. Scale bars: 100 μm. (C, D) Representative images and quantitative analysis of EdU proliferation assays for MSCs and RC-MSCs-CMDiI, demonstrating that labeling MSCs with RC and CMDiI did not affect cell proliferation. No statistically significant difference was observed between the two groups in EDU proliferation. The cell experiments were repeated three times. Scale bars: 100 μm. All data are expressed as mean ± SD and were analyzed by independent samples *t*-test. MSCs: Mesenchymal stromal cells; ns: not significant; PB: Prussian blue; RC: Ruicun; RC-MSCs-CMDiI: mesenchymal stromal cells labeled by Ruicun and CMDiI.

To determine the efficiency of labeling MSCs with SPIONs agents, we assessed the intracellular uptake of iron in MSCs using PB staining after 24 hours of RC incubation (**Figure 2A**). We observed that nearly all MSCs were labeled with RC. To determine the efficiency and status of CMDiI-labeled RC-MSCs, we used immunofluorescence to label the membrane protein marker CD90 on MSCs. The colocalization rate of CMDiI with CD90 on RC-MSCs was 100% (**Figure 2B**), and the labeling process did not affect the morphology (**Figure 2B**) or proliferation capacity (**Figure 2C** and **D**) of the RC-MSCs.

### Transplantation of mesenchymal stromal cell or ruicun-labeled mesenchymal stromal cells improved neural function in a semi-transected acute spinal cord injury beagle dog model

**Figure 3:**
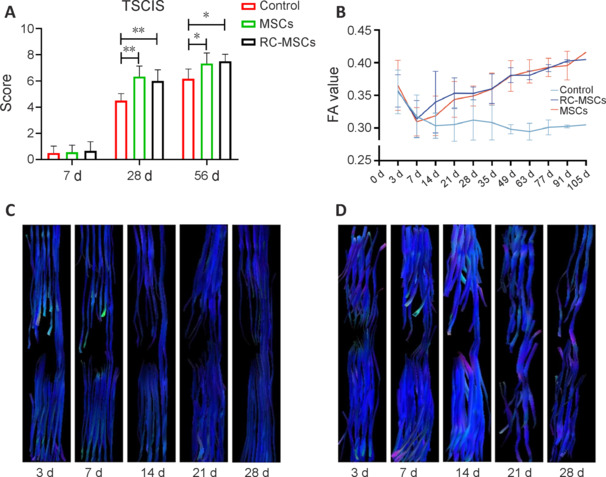
Assessment of functional recovery with TSCIS and DTI. (A) Locomotion recovery was assessed using TSCIS at 7, 28, and 56 days post-surgery. Data are presented as mean ± SD. *P < 0.05, **P < 0.01 (one-way analysis of variance followed by Tukey’s *post hoc* test). (B) Fiber bundle injury and repair were evaluated with FA. Data are presented as mean ± SD. (C, D) The three-dimensional morphological characteristics of the spinal cord fiber bundle were observed with DTT in the control group (C) and RC-MSCs group (D). The white matter fiber tract lesion appeared gradually repaired and narrowed in the RC-MSCs group, but not in the control group. DTI: Diffusion tensor imaging; DTT: diffusion tensor tractography; FA: fractional anisotropy; RC-MSCs: mesenchymal stromal cells labeled by Ruicun; TSCIS: Texas spinal cord injury scale.

To evaluate the recovery of motor function in the dogs, we use the TSCIS. As shown in **Figure 3A**, the TSCIS of the control group was slightly increased to 5.80 ± 0.84 at 56 days post-surgery compared to day 7. Compared with control group, the MSCs and RC-MSCs groups showed functional restoration in ASCI dogs at 8 weeks after treatment. There was no statistical difference in TSCIS between the MSCs and RC-MSCs groups, indicating that RC-MSCs did not affect repairability.

### Diffusion tensor imaging analysis of acute spinal cord injury in beagle dogs

In the DTI analysis, the FA of all ROIs in the control group decreased with time, whereas the FA of injured areas in the MSCs and RC-MSCs groups slowly increased with time. After the injury, the FA decreased and there were no differences between days 3, 7, 14, 21 and 28 in the control group (*F* = 2.087; *P* = 0.113). In the MSCs and RC-MSCs groups, there were within-group differences in FA between days 3 and 7 (MSCs: *P* < 0.001; RC-MSCs: *P* < 0.001), and days 14 and 21 (MSCs: *P* < 0.001; RC-MSCs: *P* < 0.01), but there was no differences between days 7 and 14 (MSCs: *P* = 0.614; RC-MSCs: *P* = 0.979), or days 21 and 28 (MSCs: *P* = 0.428; RC-MSCs: *P* = 0.754). The three groups showed no differences in FA on days 3, 7, and 14 (day 3 *vs*. day 7: *F* = 0.502, *P* = 0.615; day 3 *vs.* day 14: *F* = 0.622, *P* = 0.550; day 7 *vs.* day 14: *F* = 1.928, *P* = 0.180). At 21 and 28 days, the FA of the MSCs and RC-MSCs groups was higher than that of the control group (MSCs *vs.* control: *P* < 0.05; RC-MSCs *vs*. control: *P* < 0.01). There were no differences between the MSCs and RC-MSCs groups in FA at any time point (all *P* > 0.05; **Figure 3B**). RC labeling of MSCs did not impact the spinal cord repair in our ASCI model. DTT showed that white matter fiber tracts were broken and sparse on day 3 after injury in all dogs. The fiber tract damage gradually increased with time in the control group, and the fibers completely lost their normal configuration and structure on day 21 (**Figure 3C**). In the MSCs and RC-MSCs groups, the lesion range of white matter fiber tracts did not increase further on day 7, and appeared gradually repaired and narrowed after day 14 (**Figure 3D**). TSCIS showed that the motion and sensory function of dogs in the MSCs and RC-MSCs groups recovered with time, whereas no recovery was observed in the control group. Pearson’s linear correlation analysis showed that TSCIS was positively correlated with FA in the ASCI treatment model (MSCs: *r* = 0.866, *P* < 0.001; RC-MSCs: *r* = 0.912, *P* < 0.001).

### Use of T2*WI to view iron oxide *in vivo*

**Figure 4:**
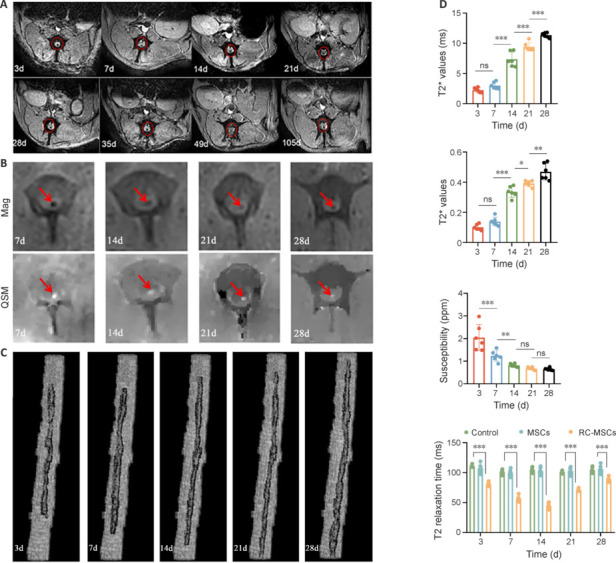
MRI assessment of RC-MSCs in ASCI Beagles. (A) Areas of low signal (red circles) were observed in axial T2*-weighted images. The volume and strength of the intramedullary low signal decreased slowly over time. (B) Axial images of the RC-MSCs group from the amplitude plot (low signal, red arrows) and magnetic susceptibility distribution plot (high signal, red arrows) in the center of the ASCI. The volume and strength of the intramedullary low signal decreased slowly over time. (C) Reconstruction images of the injured spinal cord and low signals (corresponding region of the RC-MSCs) with T2*-weighted images. These images indicated that RC-MSCs migrated to the head and tail of the injured spinal cord. (D) Data from the RC-MSCs group indicated a loss of iron signal and an increase in T2*, rT2* and T2 values, and the QSM value of the injured spinal cord decreased over time. Data are presented as mean ± SD (*n* = 6). *P < 0.05, **P < 0.01, ***P < 0.001 (one-way analysis of variance followed by the least significant difference test). ASCI: Acute spinal cord injury; MRI: magnetic resonance imaging; ns: not significant; QSM: quantitative susceptibility mapping; RC-MSCs: Mesenchymal stromal cells labeled by Ruicun.

**Figure 5:**
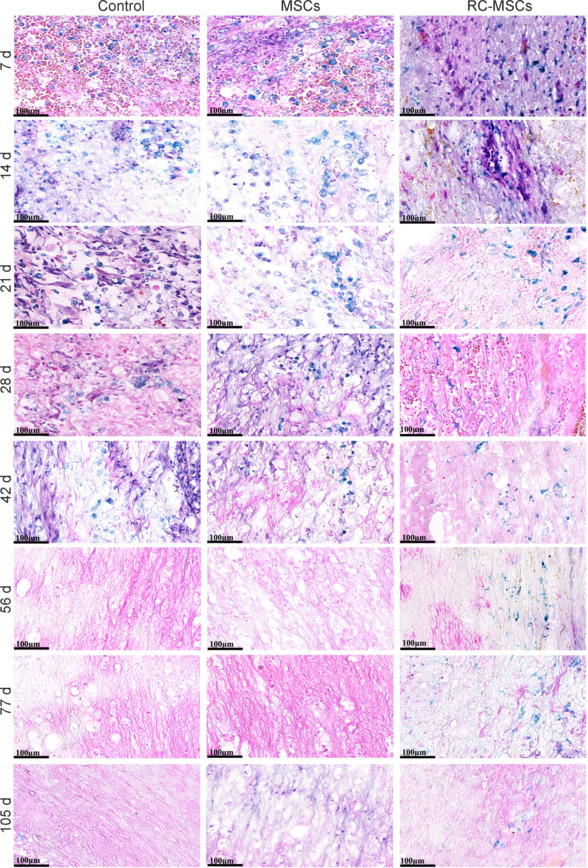
Transplanted RC-MSCs in the spinal cord of ASCI dog model. PB and eosin double staining showed that all three groups had round-like blue particle-rich cells, and only the RC-MSCs group had spindle-like blue-particle rich cells, in the postoperative period up to 42 days. The morphology and distribution indicated that the blue particle-positive cells in the MSCs and control groups were likely macrophages. At 56 days after transplantation, blue particles were found in the interstitium of the spinal cord but not in cells in the RC-MSCs group. Scale bars: 100 μm. ASCI: Acute spinal cord injury; MSC: mesenchymal stromal cell; PB: Prussian blue; RC-MSCs: mesenchymal stromal cells labeled by Ruicun.

Low signal areas were observed in T2W and T2*W images at various time points in the RC-MSCs group beginning on day 3 after transplantation. From days 7 to 105, the volume of intramedullary low signal first increased and then decreased slowly, and the signal was observed on day 105 in T2*W images (**Figure 4A**). PB staining confirmed that the blue-stained iron oxide in the intramedullary T2* low signal region was in the interstitium of the spinal cord on both sides of the injury on day 105. Pathological and immunohistochemical analyses together confirmed that the hypointense signal was the RC-labeled viable MSCs for 4 weeks, which were traced up to day 105 *in vivo* (**Figure 5**).

### Quantitative analysis of ruicun-labeled mesenchymal stromal cells

T2* mapping and QSM achieved *in vivo* tracing of RC-MSCs and nano iron quantification. In the amplitude plot (Mag plot) and magnetic susceptibility distribution plot (QSM plot), the characteristic iron oxide signals were observed in the region corresponding to the low signal area of T2* in the intramedullary region. In the amplitude plot, RC-MSCs showed a low signal, and in the magnetic susceptibility plot, RC-MSCs showed a high signal (**Figure 4B**). Within 28 days after transplantation, histopathological analysis of T2* and QSM indicated that the number of RC-MSCs migrating to the head and tail of the injured cells was increased, and the number of cells per unit volume was decreased (**Figure 4C**). RC located in the cells was used for quantification and as *in vivo* tracers of labeled cells.

### Trends of T2*, rT2*, QSM, and T_2_ over time in the ruicun-labeled mesenchymal stromal cells group

After RC-MSC transplantation, T2* and rT2* values of the damaged layer in the RC-MSCs group increased with time, and the rate of increase gradually decreased. There were significant differences in T2* relaxation time and rT2* between days 7 and 14 (T2* relaxation time: *P* < 0.001; rT2*: *P* < 0.001), days 14 and 21 (T2* relaxation time: *P* < 0.001; rT2*: *P* < 0.05), and days 21 and 28 (T2* relaxation time: *P* < 0.001; rT2*: *P* < 0.001), whereas there were no differences between days 3 and 7 (T2* relaxation time: *P* = 0.090; rT2*: *P* = 0.066). After RC-MSC transplantation, the QSM at the central layer of the lesion in the RC-MSCs group decreased with time, and the rate of decrease was gradually decreased. There were statistically significant differences in QSM between days 3 and 7 (*P* < 0.001), and between days 7 and 14 (*P* < 0.05), but there were no differences between days 14 and 21, or 21 and 28 (day 14 *vs.* day 21: *P* = 0.413, day 21 *vs*. day 28: *P* = 0.863). Pearson’s linear analysis was conducted for T2*, rT2*, and QSM. T2* relaxation time was positively correlated with rT2* (*r* = 0.924, *P* < 0.001). T2* and rT2* were negatively correlated with QSM (T2*: *r* = −0.764, *P* < 0.001; rT2*: *r* = −0.779, *P* < 0.001). There was a difference within 21 days on T2WI. RC-MSCs showed a hypointense signal effect, whereas unlabeled MSCs showed an isointense signal when compared with the adjacent area of the spinal cord. Labeled and unlabeled MSCs showed statistical differences in T2 relaxation times on days 3, 7, 14, 21, and 28 (**Figure 4D**).

### The effectiveness of magnetic resonance imaging in tracking ruicun-labeled mesenchymal stromal cells was demonstrated through histopathological analysis

Histological assessment showed the results of PB and eosin double staining of spinal cord sections at different time points. Within 42 days post-operation, all three groups exhibited round iron-rich cells, and only the RC-MSCs group displayed spindle-shaped iron-rich cells (**Figure 5**).

**Figure 6:**
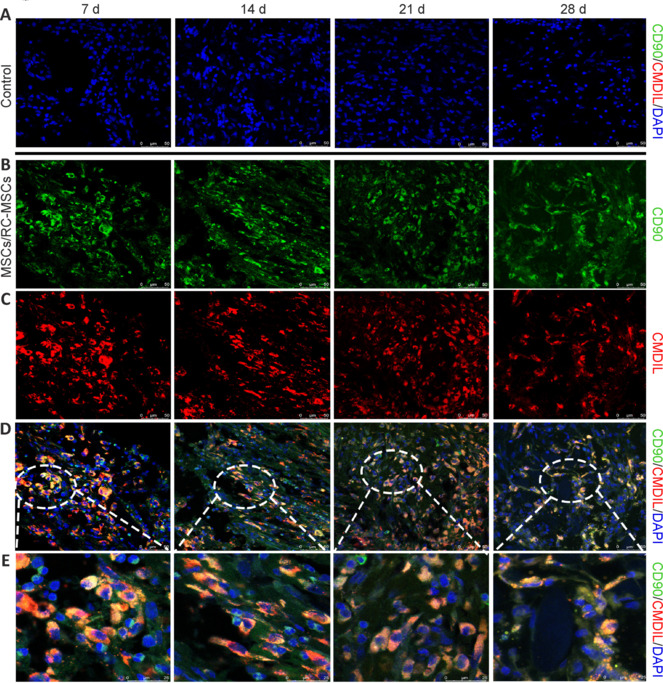
Confocal images of MSCs at 7, 14, 21, and 28 days post-operation. (A) Representative immunofluorescence images of SCI in the control group at 7, 14, 21, and 28 days post-operation. No specific green fluorescence (CD90, Alexa Fluor 488) or red fluorescence (CMDiI) of MSCs was observed, only blue nuclear staining was detected. (B) Immunofluorescence images of spinal cord tissue showing specific antibody staining for CD90 on MSCs. (C) Fluorescence imaging of MSCs in spinal cord tissue with CMDiI labeling. (D) Merged confocal images of DAPI (blue), specific antibody CD90 (green), and CMDiI (red) staining in spinal cord tissue. (E) Enlarged view of D. (B–E) Representative images of stained spinal cord tissue in the cell transplantation group at 7, 14, 21, and 28 days post-operation. The results showed that the transplanted MSCs in the MSCs group and RC-MSCs group remained viable for up to 28 days, and over this period, the number of viable MSCs decreased with time. Scale bars: 50 μm in A–D; 25 μm in E. DAPI: 4′,6-Diamidino-2-phenylindole; MSCs: mesenchymal stromal cells; RC-MSCs: mesenchymal stromal cells labeled by Ruicun.

**Figure 7:**
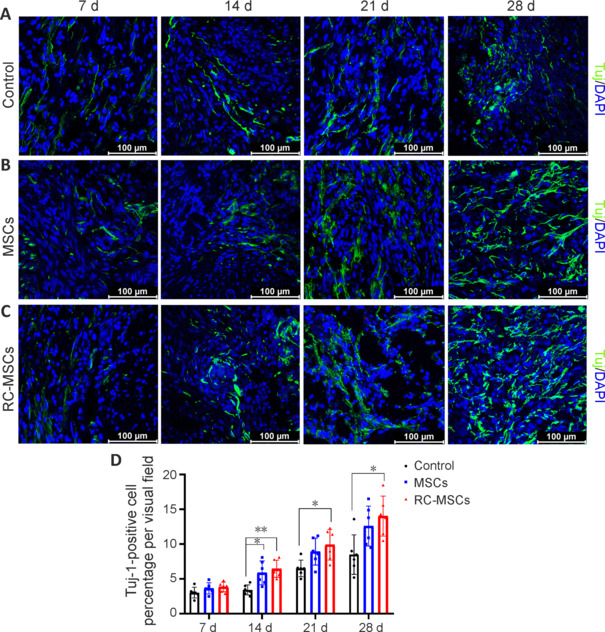
MSCs and RC-MSCs treatments promote nerve regeneration in ASCI beagle model. (A–C) Representative images of immunostaining for Tuj-1 (neurons; Alexa Fluor 488, green). The numbers of Tuj-1-positive cells in the peri-lesion area in the MSCs and RC-MSCs groups were higher than that in the control group for 28 days. During the 28-day observation period, there was a trend for the mean percentage of Tuj-1-positive cells at the SCI site in the RC-MSCs group to be higher than that in the MSCs group at all four time points, but these differences did not reach statistical significance. Scale bars: 100 μm. (D) The number of Tuj-1-positive cells in the spinal cord tissue at different time points is presented as mean ± SD (*n* = 6). *P < 0.05, **P < 0.01 (one-way analysis of variance followed by Tukey’s *post hoc* test). ASCI: Acute spinal cord injury; DAPI: 4′,6-diamidino-2-phenylindole; MSCs: mesenchymal stromal cells; RC-MSCs: mesenchymal stromal cells labeled by Ruicun; Tuj-1: neuron-specific class III beta-tubulin.

To verify MSC survival around the injured spinal cord tissue, we performed tricolor immunofluorescent co-staining analysis of DAPI/CMDiI/CD90 on spinal cord tissue. Positive cells were observed in both the RC-MSCs and MSCs groups at all time points within 28 days, whereas no positive cells were seen in the control group (**Figure 6**). To demonstrate the effect of MSC transplantation on SCI repair, we performed immunofluorescence detection of the neuronal marker Tuj-1 on spinal cord tissue at different time points within 28 days. Tuj-1 expression in the MSCs and RC-MSCs groups was higher than that in the control group on days 7, 14, 21, and 28. Quantitative analyses of Tuj-1-positive cells conducted at the same four time points indicated an increasing trend in the average proportion of Tuj-1-positive cells at the spinal cord lesion site in the RC-MSCs group compared with the MSCs group; however, this increase did not achieve statistical significance (**Figure 7**).

## Discussion

Both RC-MSCs transplantation and MSCs transplantation positively affected functional restoration in the ASCI model compared with the control group in the present study, which provides important information for the clinical translation of RC-based MSC transplantation studies. Additionally, RC-labeling allowed for detailed tracking of MSC transplantation in the host, which cannot be obtained when using non-labeled MSCs. To determine MSC survival at different time points post-transplantation, we performed co-labeling with the CD90 membrane protein (green fluorescence) and CMDiI (red fluorescence). Only when these two fluorescence signals overlapped did we consider the MSCs to be viable. This method effectively identifies most of the surviving MSCs. A persistent and strong iron signal after RC-MSC transplantation indicated that transplanted MSCs survived for at least 28 days in the host, suggesting their effectiveness for repair in ASCI.

RC labeling allowed for observation of MSC dynamics 3 days after transplantation. TSCIS did not differentiate the MSCs group from the RC-MSCs group. Spinal cord fiber bundle repair was observed using MRI based on the morphology, signal intensity, and area of the spinal cord within 28 days after implantation, as evaluated by TSCIS. In this study, RC labeling was performed through “off-label” use of the iron supplement, which was approved by the National Medical Products Administration.

To the best of our knowledge, *in vivo* tracing of single iron-labeled MSCs by MR has been achieved in experimental studies, but because of the short observation period, long-term biological behavior was not tracked (Zohar et al., 1997). Paramagnetic substances can destroy the uniformity of the local magnetic field, causing adjacent protons to lose phase coherence in a concentration-dependent manner, shortening the transverse relaxation time (T2 and T2*), and resulting in a decrease in T2 and T2* relaxation time (Wu et al., 2012). Common paramagnetic substances in human tissues include ferritin and hemosiderin. Compared with the T2 relaxation time, the T2* relaxation time was more sensitive to the inhomogeneity of the magnetic field and easier to identify changes in the magnetic susceptibility of tissues (SPIONs produced a “blooming effect” in which the hypointense MRI signal was amplified, which was larger than that of the iron-labeled graft). In this study, 3 days after RC-MSC transplantation, low signal areas on T2* mapping were observed on both sides of the injured spinal cord in the RC-MSCs group. Combined with the immunohistochemistry results, these results indicated that the MSCs that migrated from the injured area to the head and tail of the spinal cord reached the detectable number by MRI, confirming that MSCs migrated to the injured site in their characteristic manner. Blue-stained iron by PB staining was always observed by the low signal region of T2* mapping. Blue iron nanoparticles were still found in cells at 49 days after transplantation. RC was found in the interstitium of the spinal cord but not in cells at 105 days after transplantation, showing a low signal region on MRI. MSCs were detected in the RC-MSCs and MSCs groups within 4 weeks after transplantation, whereas no MSCs were detected in the control group. Therefore, we concluded that the low signal areas in T2* mapping were the implanted RC-MSCs in the RC-MSCs group. Because cell proliferation, differentiation, and death occur after 28 days, whether the low signal area indicates RC-MSCs needs further verification.

Quantification of the iron-labeled graft would have significant impact in clinical practice, as would information on the grafted cell dynamics for targeted therapy. Conventional QSM sequences are unable to quantify high iron concentration due to significant T2* shortening, leading to rapid signal decay (Lu et al., 2018). In this study, novel UTE-QSM sequences were applied to detect and quantify iron, and the results suggested that MSC implants survived, migrated to the target areas, and integrated with the host. RC was stable in MSCs over 4 weeks. To date, the most common clinical application of T2* mapping is the quantitative analysis of iron deposition in the liver and heart (Mokhtar et al., 2016; Dekkers and Lamb, 2018). Previous literature reported that the T2* relaxation time was negatively correlated with the tissue iron content, indicating that the higher the iron content was, the lower the T2* relaxation time. A T2* relaxation time of < 20 ms indicated cardiac iron deposition (Wood et al., 2004). Other studies confirmed that QSM was significantly correlated with the local iron content in brain tissue (Zheng et al., 2013), laying a theoretical foundation for the quantitative analysis of brain iron content using QSM technology. QSM technology has been widely used in the diagnosis and evaluation of cerebral iron deposition-related neurological diseases, such as cerebral hemorrhage (Sun et al., 2018) and Alzheimer’s disease (Kim et al., 2017). Previous MR tracer studies of RC-MSCs focused on MRI observations, and no quantitative analysis was conducted. To the best of our knowledge, this study is the first to use UTE-QSM to quantify RC-MSCs, and the RC-MSC dynamics over time in hosts were explored by quantitative parameters. The rT2* was more stable than the T2* without interference from background noise. T2* and rT2* of the RC-MSCs group increased with time whereas the QSM decreased, indicating that the iron concentration in the injured area gradually decreased with time. These changes may have been caused by RC-MSCs migration, cerebrospinal fluid flow, and macrophage clearance. However, it should be noted that MR techniques cannot directly measure the iron concentration in tissues. We demonstrated the ability of UTE-QSM to quantify iron concentration, which showed a strong linear relationship. Furthermore, the UTE-QSM was more sensitive to the changes in tissue magnetic susceptibility.

The FA was found to be a sensitive indicator of spinal cord axon injury. It has been demonstrated that FA correlates well with histopathological indicators and has been used for early, quantitative, and noninvasive evaluation of fiber bundle injury and repair (Cohen et al., 2017; Yung et al., 2019). Our findings showed that FA in the control group continuously decreased over time after ASCI. From day 14, the downward trend gradually slowed down to a plateau, which was consistent with previous research results (Ellingson et al., 2008). FA in the MSCs and RC-MSCs groups initially decreased and then gradually increased from day 14, and was significantly higher than that of the control group. This may be explained by the repair of the axon structure of the damaged fiber bundle by the transplanted MSCs, restricting the axial diffusion of water molecules. Moreover, FA exhibited a good correlation with the TSCIS. The TSCIS is the most widely used motor function evaluation method in beagle models with ASCI and has a good correlation with ASCI severity. The high correlation between FA and TSCIS indicated that FA could be used as an imaging index for quantifying spinal nerve function after ASCI. As FA measures the amount of diffusion asymmetry within a voxel, which reflects the integrity of fiber tracts, it is not as sensitive to the magnetic field disturbances caused by the labeled cells as T2* (Fang et al., 2018). DTT can visually display the three-dimensional morphological characteristics of the spinal cord fiber bundle. The spinal cord fiber bundles were damaged after ASCI, and the degree of injury worsened over time. Finally, the normal structure was completely lost in the control group. We speculated that continuous secondary injury led to the progressive destruction of the residual spinal neurons. In contrast, in the MSC transplantation groups, the white matter fibers of the spinal cord began to recover 7 days after ASCI.

In this study, we aimed to validate the accuracy of FA, an MRI parameter, for evaluating neural regeneration post-ASCI. Using spinal cord specimens from a beagle model, harvested at specific time points after injury, we conducted anti-Tuj-1 immunocytochemical staining to investigate the impact of RC-MSCs on neuronal differentiation. We also assessed the efficacy of imaging modalities in monitoring these intricate cellular processes. The results showed evidence of white matter fiber repair in the spinal cord as early as 7 days post-ASCI in the MSC transplantation groups. At 28 days post-transplantation, MSCs were consistently detected within the host tissue, indicating their sustained functional presence. Tuj-1 expression was significantly increased in the MSC transplantation group compared with that in the control, suggesting enhanced neuronal gene expression within the injury site attributable to MSC transplantation. The observed synergistic effect, characterized by the differentiation of transplanted stromal cells into neurons and the recruitment of host neurons to the injury site via paracrine mechanisms, highlights the potential of MSC transplantation as a viable therapeutic strategy for ASCI.

In the experimental group where cells were transplanted following treatment with SPIONs RC, there was a trend towards increased Tuj-1 mean fluorescence intensity in the ASCI region than that in the group receiving only MSCs. However, due to the limited sample size, this difference was not statistically significant. This observation suggests that SPIONs function not only as a noninvasive imaging tool for *in vivo* cell fate mapping but also potentially enhance neuronal cell genesis. These findings align with those of Dai et al. (2019), which demonstrated the synergistic application of SPIONs and an external magnetic field facilitated neural differentiation in mouse embryonic stem cells. Furthermore, Mohammadalizadeh et al. (2022) reported that integrating SPIONs into poly (lactic-co-glycolic acid) nanofibers augmented the expression of neuronal markers, including differentiation in stem cells. The mechanism involves organelle-mediated release of iron ions into the cytoplasm from iron oxide nanoparticles, hypothesized to stimulate neurite outgrowth. This integrated approach provides valuable insights into enhancing neural regeneration and offers potential avenues for future therapeutic interventions in SCI.

Our study has some limitations. Firstly, our animal experiment did not include a sham surgery group, which neglects the impact of the surgery itself on the beagle SCI model. Secondly, MRI techniques such as DTI and QSM are susceptible to motion. Therefore, interference from factors such as respiratory movement and aortic pulsation during the scan may cause deviations in the measured values. Lastly, the QSM sequence settings and post-processing methods are primarily designed for the human head, and their accuracy in application to the animal spinal cord requires further verification.

The findings of the present study indicated that MSC transplantation repaired damaged spinal cord fibers and restored nerve function in an ASCI model. Multimodal MRI was valuable in the dynamic evaluation of morphological and neurological changes after ASCI in beagles and dynamically evaluated a series of biological behaviors of transplanted MSCs. The quantitative parameters of DTI were correlated with the injury and repairability of spinal cord white matter fiber tracts, and the consistency between FA and pathological staining data allowed for more comprehensive evaluation of the integrity of spinal cord white matter fiber tracts after ASCI and the repairability of fiber tracts by transplanted stromal cells. T2*WI mapping showed a good correlation with QSM, and the quantitative values were stable and reliable. In ASCI, RC indirectly traced the distribution and characteristics of MSCs, and potentially has the ability to enhance the restoration of neural function after SCI.

## Data Availability

*No additional data are available*.
